# Disparate Effects of Diabetes and Hyperlipidemia on Experimental Kidney Disease

**DOI:** 10.3389/fphys.2020.00518

**Published:** 2020-06-03

**Authors:** Anna M. D. Watson, Eleanor A. M. Gould, Sarah C. Moody, Priyadharshini Sivakumaran, Karly C. Sourris, Bryna S. M. Chow, Audrey Koïtka-Weber, Terri J. Allen, Karin A. M. Jandeleit-Dahm, Mark E. Cooper, Anna C. Calkin

**Affiliations:** ^1^Central Clinical School, Monash University, Melbourne, VIC, Australia; ^2^Baker Heart and Diabetes Institute, Melbourne, VIC, Australia; ^3^German Diabetes Centre (DDZ), Leibniz Centre for Diabetes Research at Heinrich Heine, University Dusseldorf, Dusseldorf, Germany

**Keywords:** diabetes, renal disease, lipids, cholesterol, albuminuria

## Abstract

It is well established that diabetes is the major cause of chronic kidney disease worldwide. Both hyperglycemia, and more recently, advanced glycation endproducts, have been shown to play critical roles in the development of kidney disease. Moreover, the renin-angiotensin system along with growth factors and cytokines have also been shown to contribute to the onset and progression of diabetic kidney disease; however, the role of lipids in this context is poorly characterized. The current study aimed to compare the effect of 20 weeks of streptozotocin-induced diabetes or western diet feeding on kidney disease in two different mouse strains, C57BL/6 mice and hyperlipidemic apolipoprotein (apo) E knockout (KO) mice. Mice were fed a chow diet (control), a western diet (21% fat, 0.15% cholesterol) or were induced with streptozotocin-diabetes (55 mg/kg/day for 5 days) then fed a chow diet and followed for 20 weeks. The induction of diabetes was associated with a 3-fold elevation in glycated hemoglobin and an increase in kidney to body weight ratio regardless of strain (*p* < 0.0001). ApoE deficiency significantly increased plasma cholesterol and triglyceride levels and feeding of a western diet exacerbated these effects. Despite this, urinary albumin excretion (UAE) was elevated in diabetic mice to a similar extent in both strains (*p* < 0.0001) but no effect was seen with a western diet in either strain. Diabetes was also associated with extracellular matrix accumulation in both strains, and western diet feeding to a lesser extent in apoE KO mice. Consistent with this, an increase in renal mRNA expression of the fibrotic marker, fibronectin, was observed in diabetic C57BL/6 mice (*p* < 0.0001). In summary, these studies demonstrate disparate effects of diabetes and hyperlipidemia on kidney injury, with features of the diabetic milieu other than lipids suggested to play a more prominent role in driving renal pathology.

## Introduction

Diabetic kidney disease is the most common cause of end stage kidney disease worldwide (Thomas et al., 2016). Moreover, the presence of kidney disease in individuals with diabetes increases their risk of cardiovascular disease and death (Borch-Johnsen and Kreiner, 1987; de Boer et al., 2009; Ninomiya et al., 2009). The prevalence of diabetic kidney disease has increased in line with the increasing prevalence of diabetes, despite the use of agents that lower plasma glucose levels and inhibit the renin-angiotensin-aldosterone system (RAAS), suggesting that other pathways may also impact on the development of this condition (de Boer et al., 2011). Indeed, diabetes is associated with a myriad of metabolic abnormalities in addition to hyperglycemia and activation of the RAAS. These include elevated levels of advanced glycation endproducts (AGEs), reactive oxygen species (ROS), cytokines, growth factors and various other pro-inflammatory factors, which have all been demonstrated to play a role in the development of diabetic kidney disease (Wada and Makino, 2013; Lindblom et al., 2015; Zhuang and Forbes, 2016). We, and others have shown that modulation of these pathways attenuates key features of experimental diabetic kidney disease (Lassila et al., 2004, 2005; Jandeleit-Dahm et al., 2005; Jha et al., 2014; Oguiza et al., 2015). Elevated lipid levels are commonly observed in the setting of diabetes, and can act in a signaling capacity, modulate cell structure and can provide energy (Ertunc and Hotamisligil, 2016; Warraich and Rana, 2018). Moreover, they have been shown to modulate many of the abovementioned pathways associated with kidney disease (Phillips et al., 2002; Glass and Olefsky, 2012). Interestingly, feeding of a high fat diet to mice in conjunction with administration of low-dose streptozotocin is a model increasingly used to mimic type 2 diabetes and its complications (Tate et al., 2019).

Established benefits of the lipid modulating agents, hydroxymethylglutaryl-CoA reductase (HMGCR) inhibitors, better known as statins, have been demonstrated in the setting of experimental diabetic nephropathy (Bruder-Nascimento et al., 2016; Kim et al., 2016). In human diabetic kidney disease, positive findings have been observed with statins, although this is not a universal finding (Colhoun et al., 2009; Abe et al., 2011; de Zeeuw et al., 2015). de Zeeuw et al. (2015) demonstrated that greater lipid lowering was not associated with greater renoprotection and Abe et al. (2011) demonstrated that renoprotection with rosuvastatin was independent of lipids levels. This raises the possibility that the renoprotective actions of statins may in part be attributable to their pleiotropic actions, including anti-inflammatory and anti-oxidative effects. Indeed, our previous studies have demonstrated renoprotective effects of statins in the absence of changes in lipid levels in experimental models of kidney disease both with, and without diabetes (Giunti et al., 2010). Thus, it has been difficult to ascertain the direct effects of lipids on the development and progression of diabetic kidney disease.

Apolipoprotein (apo) E gene deletion in mice results in chronic hypercholesterolemia that exhibits limited responsiveness to treatment with PPARα agonists or HMG-CoA reductase inhibitors (Calkin et al., 2006, 2008). Although best known as a model of aortic plaque formation, the apoE knockout (KO) mouse is also a model of progressive renal injury (Wen et al., 2002). When induced with diabetes via the beta cell toxin, streptozotocin, these mice exhibit accelerated atherosclerosis and kidney disease (Candido et al., 2002; Lassila et al., 2004). Therefore, we utilized the apoE KO mouse model to investigate the potential for a direct causal role of lipids on the progression of kidney disease.

## Materials and Methods

### Experimental Groups

Six-week old male C57BL/6 and apoE knockout KO mice (backcrossed 20 times to a C57BL/6 background; Animal Resource Centre, Canning Vale, WA, Australia) were used in this study. Mice were housed at the Precinct Animal Centre, Baker Heart and Diabetes Institute. C57BL/6 and apoE KO mice were randomized to be fed of a chow diet (control), a western diet, consisting of 21% butter fat and 0.15% cholesterol (Specialty Feeds, Glen Forrest, Australia) or the induction of diabetes via intraperitoneal injection of streptozotocin in citrate buffer (“diabetes”; 55 mg/kg; MP Biomedicals, Eschwege, Germany) daily for 5 days and fed a chow diet. Mice with a blood glucose >15 mmol/L blood glucose were considered diabetic. Throughout the study mice were given access to chow or western diet as indicated as well as water *ad libitum.*

After 20 weeks of experimental diabetes or equivalent, mice were culled via intraperitoneal injection of Euthal (Delvet Limited, Seven Hills, Australia) followed by exsanguination by cardiac puncture. The kidneys were rapidly dissected, weighed and processed to paraffin or snap frozen for subsequent analysis. The protocols for animal experimentation and handling were approved by the Animal Welfare Committee of the Baker Heart Research Institute and the Alfred Hospital in accordance with the Australian National Health and Medical Research Council ethical guidelines.

### Measurement of Physiological and Biochemical Parameters

Prior to sacrifice, mice were placed in individual metabolic cages (Iffa Credo, L’Arbresele, France) for a period of 24 h. Body weight as well as food and water intake were recorded. Urine was collected for subsequent analysis (as described below). Glycated hemoglobin (Hb) was measured in whole blood obtained at the time of sacrifice, by high performance liquid chromatography (CLC330 GHb Analyzer; Primus, Kansas City, MO, United States) (Cefalu et al., 1994). Plasma total cholesterol and triglycerides were measured in samples obtained at the time of sacrifice, using a colorimetric assay (Wako Diagnostics, Richmond, VA, United States). Systolic blood pressure was assessed using the computerized non-invasive tail cuff method as previously described (Krege et al., 1995). Readings were taken by an experienced technician on conscious mice toward the conclusion of the study.

### Measurement of Kidney Function

Urinary albumin excretion was measured using a mouse albumin ELISA kit (Bethyl Laboratories, Montgomery, TX, United States) according to the manufacturer’s instructions. Plasma cystatin C levels were measured as a readout of kidney function (Song et al., 2009). Diluted plasma samples (1:250) were analyzed using a mouse cystatin C ELISA kit (Biovendor, Czech Republic) according to the manufacturers instructions.

### Mesangial Expansion

To evaluate kidney histopathology, 3μm kidney sections were stained with periodic acid-schiff (PAS) as described previously (Watson et al., 2010). Images (10 per mouse) were obtained on a Nikon Eclipse Ci microscope (Nikon, Tokyo, Japan) with a DS-Fi3 digital camera (Nikon, Tokyo, Japan) using NikonNIS Elements software (Nikon, Tokyo, Japan) and were taken under identical light conditions. The percent area of staining was assessed in a blinded manner in RGB as a percentage of the glomerular vascular tuft (Image Pro Plus, v6.0, Media Cybernetics, Rockville, MD, United States).

### RNA Isolation, cDNA Synthesis, and qPCR Analysis

RNA was extracted from kidney as previously described (Calkin et al., 2006). Briefly, RNA was isolated using TRIzol reagent (Thermo Fisher Scientific, MA, United States) and subsequently treated with DNAse to remove genomic DNA contamination according to the manufacturer’s instructions (Ambion, TX, United States). cDNA synthesis was performed using the Superscript First Strand Synthesis System (Thermo Fisher Scientific, MA, United States). Quantitative real-time RT-PCR was carried out using the Taqman System on an ABI Prism 7500 (Applied Biosystems, CA, United States). mRNA expression was normalized to 18S ribosomal RNA and expressed as fold change over C57BL/6 control mice. A Ct value >35 was considered background. For probe and primer sequences see Supplementary Table S1.

### Statistical Analysis and Data Handling

Data were analyzed by a 2-way ANOVA to test the effect of strain (C57BL/6, apoE KO) and treatment (chow, diabetes, western diet) followed by Tukey’s multiple comparison test within strains. Correlations were analyzed by linear regression. For qPCR and UAE, an individual data point was excluded if it fell outside of mean ± 2 SD. Three values were excluded as they were biologically implausible. One mouse was excluded from the diabetic C57BL/6 group due to the presence of a renal cyst. Data are expressed as mean + SEM unless otherwise stated. *p* < 0.05 was considered significant.

## Results

### Body Weight, Kidney Weights, and Blood Pressure

**FIGURE 1:**
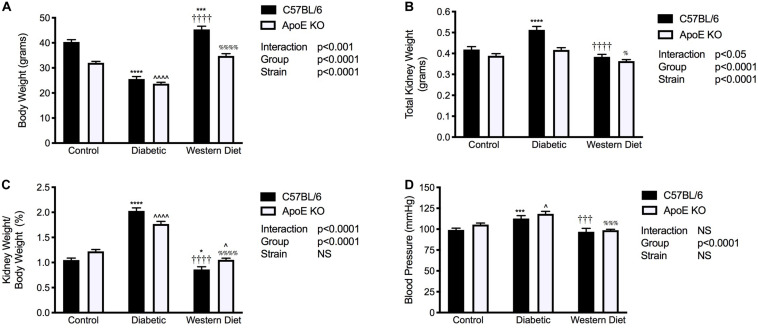
Diabetes is associated with increased kidney/body weight ratio and systolic blood pressure. **(A)** Body weight; **(B)** Kidney weight; **(C)** Kidney/body weight ratio; **(D)** Systolic blood pressure; **(A–C)**, *n* = 11–24/group; **(D)**, *n* = 8–22/group. Data represented as mean + SEM. *p < 0.05, ***p < 0.001, ****p < 0.0001 vs. C57BL/6 control; ^∧^*p* < 0.05, ^∧∧∧∧^*p* < 0.0001 vs. apoE KO control; ^†††^*p* < 0.001, ^††††^*p* < 0.0001 vs. C57BL/6 diabetic;^%^*p* < 0.05, ^%%%^*p* < 0.001, ^%%%%^*p* < 0.0001 vs. apoE diabetic.

Chow fed C57BL/6 mice weighed significantly more than their apoE KO counterparts (*p* < 0.0001; Figure 1A). For both C57BL/6 and apoE KO mice, the induction of diabetes was associated with a marked reduction in body weight compared to their chow fed counterparts (*p* < 0.0001), consistent with previous studies (Watson et al., 2012). Interestingly, no difference in body weight was observed between strains in the setting of diabetes. Feeding of a western diet was associated with a significant increase in body weight in C57BL/6 mice (*p* < 0.001), an effect not observed in apoE KO mice.

Total kidney weight was significantly elevated with diabetes only in C57BL/6 mice (*p* < 0.0001; Figure 1B) and mice fed a western diet had significantly lower kidney weight than diabetic mice for both strains. Moreover, the induction of diabetes was associated with an ~2-fold and 1.5-fold increase in kidney to body weight ratio compared to both chow and western diet fed mice for C57BL/6 and apoE KO mice respectively (*p* < 0.0001; Figure 1C). In contrast to diabetes, western diet feeding was not associated with renal hypertrophy.

Systolic blood pressure was elevated in diabetic C57BL/6 mice compared to both control and western diet fed C57BL/6 mice (*p* < 0.001; Figure 1D). Similarly, western diet feeding had no impact on blood pressure compared to chow fed mice with apoE deletion; however, diabetes was associated with an elevation in systolic blood pressure compared to both chow (*p* < 0.05) and western diet fed (*p* < 0.001) apoE KO mice.

### Metabolic Parameters

**FIGURE 2:**
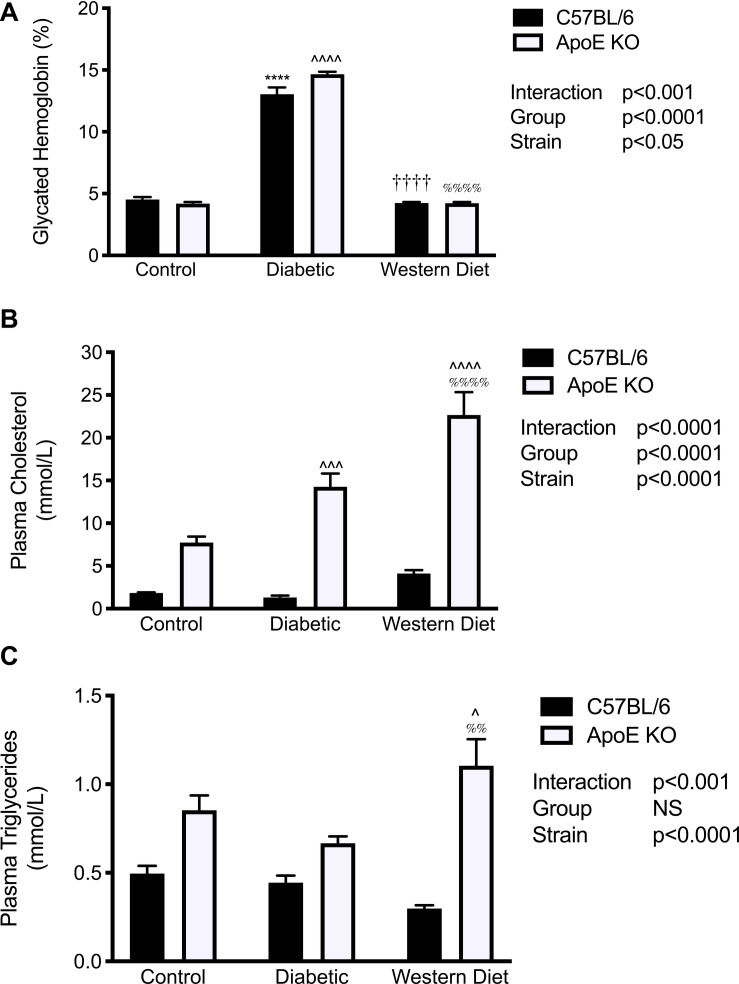
Apolipoprotein E deletion exacerbates the effect of diabetes or feeding of a western diet on plasma lipid levels. **(A)** Glycated hemoglobin; **(B)** Plasma total cholesterol levels; **(C)** Plasma triglyceride levels; **(A)** *n* = 12–23/group; **(B,C)** *n* = 4–8/group. Data represented as mean + SEM. ****p < 0.0001 vs. C57BL/6 control; ^∧^*p* < 0.05, ^∧∧∧^*p* < 0.001, ^∧∧∧∧^*p* < 0.0001 vs. apoE KO control; ^††††^*p* < 0.0001 vs. C57BL/6 diabetic; ^%%^*p* < 0.01,^%%%%^*p* < 0.0001 vs. apoE diabetic.

Twenty weeks after the induction of diabetes, both C57BL/6 and apoE KO mice diabetic mice exhibited an ~3-fold increase in glycated hemoglobin levels compared to both control and western diet fed mice (*p* < 0.0001; Figure 2A). In C57BL/6 mice, neither diabetes nor western diet feeding was associated with an elevation in plasma total cholesterol levels compared to the control group (Figure 2B). In contrast, apoE deficiency alone was associated with a greater than 4-fold increase in plasma cholesterol in mice fed a chow diet. The induction of diabetes in apoE KO mice was associated with a 2-fold increase, and feeding of a western diet a 3-fold increase in plasma cholesterol levels above that seen in control apoE KO mice. Similar to plasma cholesterol levels, plasma triglyceride levels were not significantly different across each of the three C57BL/6 groups (Figure 2C). There was a significant effect of strain on this parameter (*p* < 0.0001). Moreover, feeding of a western diet resulted in a greater elevation in plasma triglyceride levels than did the induction of diabetes in apoE KO mice (*p* < 0.01).

### Urinary Albumin Excretion

**FIGURE 3:**
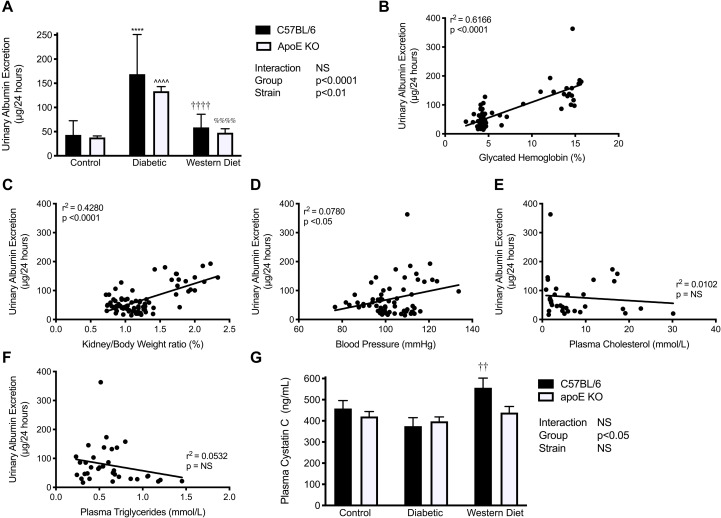
Apolipoprotein E deletion has no effect on the diabetes-associated increase in urinary albumin excretion. **(A)** Urinary albumin excretion rate (UAE); Correlation of UAE with **(B)** Glycated hemoglobin; **(C)** Kidney weight to body weight ratio; **(D)** Systolic blood pressure; **(E)** Plasma cholesterol and **(F)** Plasma triglycerides; **(G)** Plasma cystatin C levels; **(A)** *n* = 7–21/group. **(B)** *n* = 80; **(C)** *n* = 78; **(D)** *n* = 62; **(E,F)** *n* = 34; **(G)** *n* = 5–9/group. **(A)** Data represented as geometric mean + geometric standard deviation; **(B–F)** linear regression with each dot representing an individual mouse. **(G)** Data represented as mean + SEM. ****p < 0.0001 vs. C57BL/6 control; ^∧∧∧∧^*p* < 0.0001 vs. apoE KO control; ^††^*p* < 0.01; ^††††^*p* < 0.0001 vs. C57BL/6 diabetic; ^%%%%^*p* < 0.0001 vs. apoE diabetic mice.

To assess the effect of diabetes and lipids on renal damage, urinary albumin excretion was determined (UAE). Diabetes was associated with a greater than 3-fold elevation in UAE in both C57BL/6 and apoE KO mice (*p* < 0.0001; Figure 3A). In contrast, western diet feeding had no significant impact on this parameter in either strain. We then performed correlation analyses of metabolic parameters with UAE. Glycated hemoglobin was highly correlated with UAE (*r*^2^ = 0.6166, *p* < 0.0001; Figure 3B), whereas the correlation was comparatively weaker for kidney/body weight ratio, though still highly significant (*r*^2^ = 0.4280; *p* < 0.0001; Figure 3C). The correlation between UAE and systolic blood pressure was weaker again but nonetheless significant (*r*^2^ = 0.0780; *p* < 0.05; Figure 3D). In contrast, there was no association with UAE and either plasma cholesterol or triglyceride levels (Figures 3E,F). These findings highlight the lack of effect of these lipids on renal damage in this setting.

### Plasma Cystatin C Levels

Plasma cystatin C levels were significantly impacted by group (*p* < 0.05), although no significant effect of strain was observed (Figure 3G). There was a trend toward reduced cystatin C levels in diabetic mice consistent with hyperfiltration as reported previously (Thallas-Bonke et al., 2014).

### Mesangial Area

**FIGURE 4:**
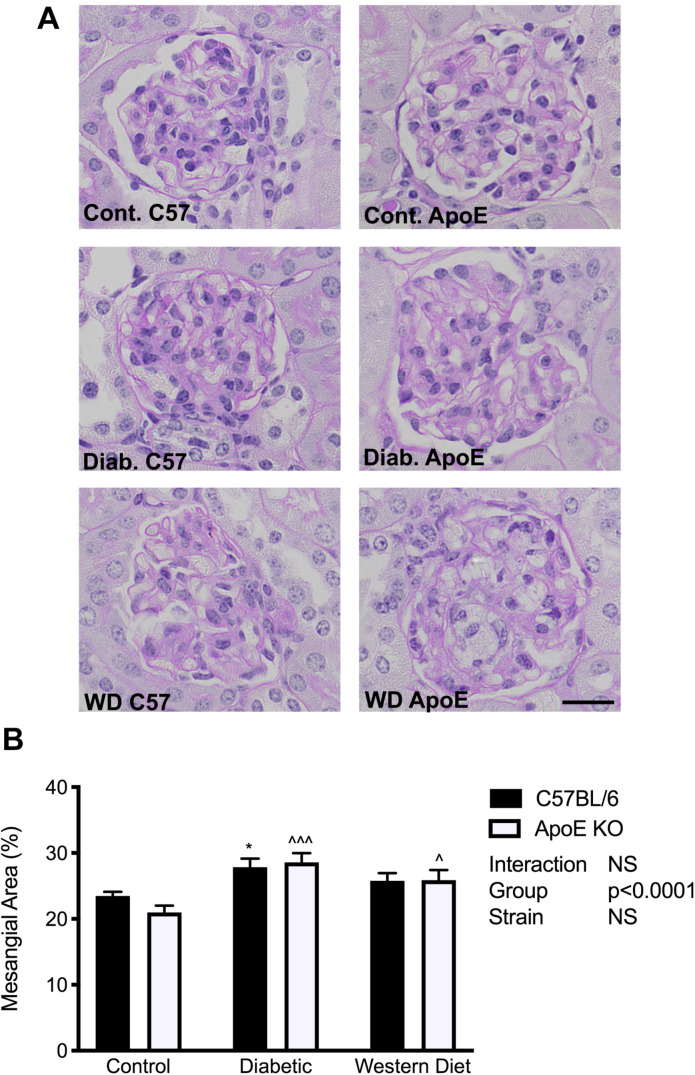
Extracellular matrix accumulation was increased with diabetes and to a lesser extent with feeding of a western diet. **(A)** Representative images of PAS staining; **(B)** Quantitation of mesangial expansion; *n* = 9–12/group. Data expressed as mean + SEM. *p < 0.05 vs. C57BL/6 control; ^∧^*p* < 0.05, ^∧∧∧^*p* < 0.001 vs. apoE KO control; Bar represents 20 μM.

To determine the effect of diabetes and lipids on extracellular matrix accumulation, mesangial area was assessed utilizing PAS-stained sections. In both strains, diabetes was associated with an accumulation of extracellular matrix proteins in the mesangium (*p* < 0.05, C57BL/6; *p* < 0.001 apoE KO) compared to chow fed mice of the same strain respectively (Figures 4A,B). Western diet feeding also promoted mesangial expansion in apoE KO mice (*p* < 0.05), although not to the same degree observed with diabetes. These data suggest a more marked effect of diabetes on extracellular matrix accumulation than lipids alone.

### Pathway Analysis

Finally, in order to examine how pathways linked to renal disease might be driving the phenotypes observed in this study, we examined the expression of markers of fibrosis and inflammation (Table 1). Analysis of mRNA expression of fibronectin, a marker of fibrosis, revealed an effect of group (*p* < 0.0001), as well as strain (*p* < 0.05). Indeed, diabetes was associated with a 2.5-fold increase in expression compared to chow fed C57BL/6 mice (*p* < 0.0001), an effect not observed in apoE KO mice. Analysis of collagen IV and connective tissue growth factor (CTGF) mRNA expression demonstrated similar trends, although the effects were not as overt as seen with fibronectin. With regard to markers of inflammatory pathways, MCP-1 mRNA expression was significantly affected by group (*p* < 0.05) but not strain, with diabetic C57BL/6 mice exhibiting a 2-fold increase compared to their control counterpart (*p* < 0.05). mRNA expression of NF-KB subunit, p65, was elevated in western diet fed apoE KO mice compared to both control and diabetic apoE KO mice (*p* < 0.05).

**TABLE 1:** Renal mRNA expression of fibrotic and inflammatory markers.

Gene	Protein	C57BL/6	ApoE KO	C57BL/6	ApoE KO	C57BL/6	ApoE KO	*p*-value	*p*-value	*p*-value
name	name	control	control	diabetic	diabetic	western diet	western diet	interaction	group	strain
*ccl2*	MCP-1	1.11 ± 0.13	1.13 ± 0.17	2.02 ± 0.36*	1.62 ± 0.39	0.73 ± 0.08^††^	1.62 ± 0.22	**0.043**	**0.013**	0.422
*col4a1*	Collagen IV	1.30 ± 0.26	1.49 ± 0.12	1.84 ± 0.29	1.38 ± 0.16	1.18 ± 0.09	1.89 ± 0.27	**0.032**	0.588	0.409
*ccn2*	CTGF	1.46 ± 0.41	0.63 ± 0.07	1.74 ± 0.23	1.15 ± 0.16	1.06 ± 0.12	1.17 ± 0.28	0.128	0.207	**0.029**
*fn1*	Fibronectin	1.11 ± 0.15	0.93 ± 0.10	2.79 ± 0.52****	1.48 ± 0.21	0.78 ± 0.09^††††^	1.02 ± 0.15	**0.008**	**< 0.001**	**0.043**
*rela*	p65	1.41 ± 0.28	1.00 ± 0.10	1.25 ± 0.17	0.72 ± 0.09	0.95 ± 0.08	1.73 ± 0.32^∧%%^	**0.003**	0.217	0.733

Monocyte chemoattractant protein (MCP)-1; Connective Tissue Growth Factor (CTGF); p65 subunit of NF-KB (p65). Data expressed as mean ± SEM. n = 10–14/group; *p < 0.05, ****p < 0.0001 vs. C57BL/6 control; ^∧^p < 0.05 vs. apoE KO control; ^††^p < 0.01, ^††††^p < 0.0001 vs. C57BL/6 diabetic; ^%%^p < 0.01 vs. apoE KO diabetic. The bolded values are *p* < 0.05.

## Discussion

Diabetes is well established to drive renal pathology, being the leading cause of chronic kidney disease worldwide (de Boer et al., 2011). A variety of mechanisms have been implicated in the progression of this condition, including oxidative stress, glycation and inflammation (Wada and Makino, 2013; Lindblom et al., 2015; Zhuang and Forbes, 2016), with the role of lipids less well defined.

In this study, we sought to establish the role of lipids as an independent driver of kidney disease. We demonstrated that deletion of apoE *per se* was associated with hyperlipidemia via elevations in plasma levels of both cholesterol and triglycerides; however, little consequent effect on urinary albumin excretion, plasma cystatin C levels or glomerular matrix accumulation were observed. Twenty weeks of diabetes was sufficient to induce renal hypertrophy, an increase in blood pressure and mesangial expansion. Furthermore, this was associated with a 3-fold increase in UAE; however, these effects were not exacerbated with apoE deletion. In contrast, despite feeding of a western diet being associated with potentiation of plasma cholesterol and triglyceride levels in apoE KO mice, only a small increase in mesangial expansion and no effect on albuminuria was observed. Overall, these data suggest that the elevation in cholesterol and triglycerides observed in our studies were not sufficient to drive overt changes in renal structure and function.

The lack of effect of apoE deletion on renal dysfunction or glomerular extracellular matrix accumulation was surprising given the findings of Lassila et al. (2004), who demonstrated that apoE deletion *per se* was associated with increased glomerular and tubulointerstitial injury. Consistent with our findings, however, they did not observe any difference in albuminuria when comparing apoE KO mice and C57BL/6 mice on a chow diet. The differences in these studies may be attributed to the fact that apoE deletion was associated with a more marked elevation in plasma cholesterol levels (1.5-fold) as well as a significant increase in blood pressure compared to control C57BL/6 mice in the study by Lassila et al., suggesting a more accelerated disease status. Indeed, Pei et al. (2016) demonstrated that a diet high in cholesterol, 1% in comparison to the 0.15% used the current study, resulted in plasma cholesterol levels more than twice what we observed in the current study. This was associated with enhanced mesangial expansion and concurrent inflammation. A plausible reason for the differences between studies with regard to blood pressure is the environment in which they were studied. Indeed, differences in environmental factors likely to vary between animal housing facilities such as caging systems and bedding can impact on gut microbiota, and have been put forward as a reason for the variability observed between preclinical studies (Franklin and Ericsson, 2017; Turner, 2018). Furthermore, gut microbiota has been shown to play a role in the regulation blood pressure (Li et al., 2017; Marques et al., 2017; Ma and Li, 2018; Toral et al., 2019). Although Lassila et al. did not observe differences in systolic blood pressure in the setting of diabetes as in the current study, this effect has been observed by others (Zhong et al., 2015; Jiao et al., 2018).

An elevation in plasma lipids alone was not sufficient to cause substantial renal damage, and this was further highlighted by the absence of a correlation between either plasma triglycerides or cholesterol levels and UAE. The fact that glycated hemoglobin was significantly correlated with UAE, highlights the previously established relationship between hyperglycemia and renal function as seen in diabetes (Chow et al., 2004). Moreover, it demonstrates that the severity of diabetes is proportional to renal dysfunction, and exemplifies the need for longitudinal assessment of diabetes status, such as glycated hemoglobin, rather than single point blood glucose measures which reflects only a single point in time. This does not necessarily implicate glucose pathways *per se* in driving changes in renal function, but merely demonstrates that one or more components of the diabetic milieu other than cholesterol or triglycerides, are likely to be driving these changes. Similar results were observed by Hammad et al. (2003) who compared wild type mice (WT) with hypercholesterolemic low density lipoprotein receptor (LDLR) apoB mRNA editing catalytic polypeptide1 (apoBec) double KO (DKO) mice. Indeed, they demonstrated that whilst STZ-diabetes was associated with albuminuria in both WT and DKO mice, no effect of hypercholesterolemia was observed in either the absence or presence of diabetes.

In the current study, diabetic C57BL/6 mice exhibited no significant elevation in plasma cholesterol or triglyceride levels yet they exhibited a marked elevation in UAE and exhibited mesangial expansion. In contrast, western diet fed apoE KO mice, which have the highest lipid burden of all groups, exhibited no change in albuminuria and only modest mesangial expansion. This further highlights the lack of a causal effect of these lipids on renal pathology in this setting. It must be noted though, that we were not able to assess the effect of overt hypertriglyceridemia. Whilst we observed significant elevations in plasma cholesterol levels, the changes in plasma triglycerides were not as marked. Individuals with type 2 diabetes commonly exhibit significant elevations in plasma triglycerides, and we were unable to recapitulate this effect in our study (American Diabetes Association, 1997). Indeed, triglyceride-rich lipoproteins have been shown to promote mesangial cell proliferation as well as increase growth factor and inflammatory cytokine secretion (Nishida et al., 1999). Furthermore, feeding of a high fat diet (HFD) consisting of 43% fat to C57BL/6 mice over a period of 29 weeks was associated with a significant elevation in plasma triglycerides and a concomitant increase in the urinary albumin to creatinine ratio and serum creatinine levels (Glastras et al., 2016). Moreover, measures of glomerulosclerosis and tubulointerstitial fibrosis were greater in HFD fed mice compared to chow fed STZ diabetic mice that did not exhibit an elevation in plasma triglyceride levels. This study raises another factor that may be influencing renal pathology, the effect of age. Whilst we observed little impact of apoE deletion *per se* on mesangial expansion, Chen et al. (2001) observed a much greater impact, albeit they studied mice at 14–16 months of age in comparison to the current study in which mice were only 6 months old. Given that renal disease is progressive, it is not surprising that age-dependent effects were observed (Remuzzi et al., 2006).

An alternate approach to assess the contribution of lipids to diabetic kidney disease is to attenuate lipid-associated pathways in the diabetic context. To that end, Falkevall et al. (2017) demonstrated that pharmacological neutralization of VEGF-B, which promotes lipid uptake, was associated with a reduction in renal lipid accumulation in STZ-diabetic and *db/db* (BKS) mice. Concomitant improvements in renal function and renal injury were observed with only a modest effect on hyperglycemia (Falkevall et al., 2017). Interestingly, the authors observed reductions in the renal accumulation of a range of lipid classes including sphinogmyelins, phosphatidylcholines, and phosphatidylethanolamines. This raises the consideration of modulating specific lipid species within the kidney itself, and their consequent effects on renal function.

In conclusion, we observed that hyperlipidemia had a mild effect on driving renal pathology, albeit in the setting of a modest elevation in plasma triglyceride levels. These findings suggest that other non-lipid factors of the diabetic milieu are likely to be playing a more important role in this setting. Had mice exhibited larger elevations in plasma lipids (Lassila et al., 2004; Pei et al., 2016), it is possible that a more severe renal pathology may have been observed. However, such marked changes in lipids would not be a true representation of that that seen in the clinical setting. Further studies utilizing lipidomic analysis would likely provide additional insight into the importance of specific lipid species not just in the circulation but also in the kidney *per se* in the setting of diabetic kidney disease.

These findings are consistent with those arising from the clinical trials and meta-analyses over the last decade. Indeed, SHARP (Haynes et al., 2014) and ALERT (Fellstrom et al., 2004) demonstrated no clear benefit of lipid lowering agents on renal disease progression, nor an association between lipid levels and progression of kidney disease (Rahman et al., 2014). Nevertheless, kidney disease is associated with a high cardiovascular burden, for which there is a clear and established benefit of lipid lowering agents (Boekholdt et al., 2014; Sabatine et al., 2017).

## Data Availability Statement

The datasets generated for this study are available on request to the corresponding author.

## Ethics Statement

The animal study was reviewed and approved by Animal Welfare Committee of the Baker Heart Research Institute and the Alfred Hospital.

## Author Contributions

AC, TA, KJ-D, and MC designed the study. AC, AW, EG, SM, PS, KS, BC, and AK-W collected and analyzed the data. AC and AW wrote the manuscript. All authors reviewed the manuscript.

## Conflict of Interest

The authors declare that the research was conducted in the absence of any commercial or financial relationships that could be construed as a potential conflict of interest.
