# Supplementary material for: Disparate Effects of Diabetes and Hyperlipidemia on Experimental Kidney Disease
Source: Front Physiol. 2020 Jun 3;11:518. doi: 10.3389/fphys.2020.00518 (PMC7283908; doi:10.3389/fphys.2020.00518)
Supplement: Supplementary file 1 [file Table_1.pdf]

Supplementary Table 1. Quantitative RT-PCR probe and primer sequences

Gene	Protein	Probe sequence	Forward Primer 5’-3’	Reverse Primer 5’-3’
Ccl2	MCP-1	AATGGGTCCAGACATA	GTCTGTGCTGACCCCAAGAAG	TGGTTCCGATCCAGGTTTTTA
Col4a1	Collagen IV	CAGTGCCCTAACGGT	GGCGGTACACAGTCAGACCAT	GGAATAGCCGATCCACAGTGA
Ccn2	CTGF	ACTGCCTGGTCCAGAC	TGGCCCTGACCCAACTATGA	CTTAGAACAGGCGCTCCACTCT
Fn1	Fibronectin	CCCCGTCAGGCTTA	ACATGGCTTTAGGCGGACAA	ACATTCGGCAGGTATGGTCTTG
Rela	p65	AGCTCAAGATCTGCCG	ACCGTGCCCCCAACACT	CAAGGCAGCTCCCAGAGTTC

Monocyte chemoattractant protein-1 (MCP-1); Connective tissue growth factor (CTGF); p65 subunit of NF-KB (p65).
`
